# Recent Trends in Enzyme Inhibition and Activation in Drug Design

**DOI:** 10.3390/molecules26010017

**Published:** 2020-12-22

**Authors:** Athina Geronikaki

**Affiliations:** Department of Medicinal Chemistry, School of Pharmacy, Aristotle University of Thessaloniki, 54124 Thessaloniki, Greece; geronik@pharm.auth.gr

It is known that enzymes are involved in many pathological conditions, such as inflammation, diabetes, microbial infections, HIV, neoplastic, neglected diseases and others. Enzyme inhibition is universally accepted as a strategy to treat the above-mentioned conditions or to elucidate the mechanism involved. In the drug design process, the design of potent enzyme inhibitors is a crucial step in the long way of drug development.

This issue is dedicated to current trends in enzyme inhibition and activation in drug design. Through this issue, you will discover the role of enzymes in many pathological conditions, the strategy used for the development of enzyme inhibitors, the targets for the development of new drugs as well as the role of computational chemistry in drug design.

One group of papers is dedicated to inhibitors of HIV-1. This virus infected over 60 million people placing AIDS as the fourth cause of death worldwide. Despite the achievements of antiretroviral therapy, the discovery of new anti-HIV medicines remains a critical task due to the fact that the existing drugs do not provide a complete cure to the patients, exhibit strong side effects, and lead to the appearance of resistant strains. Thus, there is still room for the development of novel anti-HIV-1 agents.

One hundred thirty-two benzothiazole-based thiazolidinones were designed. Based on prediction of biologically spectra of substances (PASS )prediction results and molecular docking, the selected thirty-two of the compounds were synthesized and evaluated against HIV-1 RT [1]. Twenty four of synthesized compounds showed inhibitory action equal or lower than 4 μM, while seven of them appeared to be more potent than nevirapin under conditions of the experiment. Three compounds exhibited IC50 lower than 5 nM.

Pyrimidine derivatives are reported as a new class of inhibitors of HIV-I reverse transcriptase associated ribonuclease H activity (RNase H), which constitutes an alternative promising anti-HIV-1 target. Structural optimization of pyrimidine derivatives, previously designed as anti-influenza agents, allowed the development of allosteric inhibitors of the RNase H function, with IC50 of two of them in the sub-micromolar range (IC50 = 0.8 and 0.41 μM, respectively) [2].

Tramontano et al. [3] presented the synthesis and testing of forty-four compounds based on 2-amino-6-(trifluoromethyl)nicotinic acid scaffold as promising RNase H dual inhibitors. Thirty-four of the compounds inhibited HIV-1 RT- associated RNase H function in the low micromolar range and seven of them also inhibited viral replication in cell-based assays with selectivity index up to 10. The most promising compound showed IC50 of 14 μM as an inhibitor of RNase H function and HIV-1 replication in cell-based bioassays. According to studies of the mode of action, one compound was found to be an allosteric dual-site agent inhibiting both HIV-1 RT functions.

Next, the paper deals with the human ATPase/RNA helicase X-linked DEAD-box 

polypeptide 3 (DDX3X) which is a novel therapeutic target in the fight against both infectious diseases and cancer. Its fundamental role was highlighted in the HIV-1 life cycle as a shuttle protein able to export the viral RNA from the nucleus to the cytoplasm. Thus, the designed, synthesized and testing for inhibitory action on the ATPase activity a new family of DDX3X inhibitors was reported [4]. The most promising derivative appeared to be a good inhibitor of the ATPase activity of DDX3X protein, showing also promising anti-HIV-1 activity in a reference virus-cell system without being cytotoxic.

Finally, the prediction of the interaction of drug-like compounds with multiple targets for HIV-1 treatment using a ligand-based drug design approach was presented. The authors created an overall training set including data from three databases NIAID, ChEMBL, and integrity for the development of the (Q)SAR models with the broadest coverage of chemical space. These (Q)SAR models were used for creating the web application that predicts anti-HIV activity [5].

Another subject highlighted in this issue is SARS-CoV-2, which has caused a pandemic across the globe, where countries are being challenged in ways they have never been before. Tarasova et al. [6] demonstrated the helpfulness of text and data mining techniques to find human proteins involved in similar pathological processes involving well-known and novel viruses. This technique was used to identify possible molecular pathways that can be shared by SARS-CoV-2 and HIV-1 viruses. By identifying a list of 46 targets that can be crucial for the development of infections caused by SARS-CoV-2 and HIV-1 it was shown that SARS-CoV-2 and HIV share some molecular pathways implicated in inflammation, immune response and cell cycle regulation. 

Eleftheriou et al. [7] compared the 3D structure of SARS-CoV-2.main protease with the 3D structures of seven proteases which are drug targets. Furthermore, thirty-four approved and on-trial protease inhibitors were docked to the SARS-CoV-2 protease. The comparison of the 3D structure alignment of the SARS-CoV-2 main protease with the HIV-1 and HCV viral proteases as well as with the human proteases DPP-4, thrombin, coagulation Factor Xa, renin and ACE revealed greater similarity with the HCV protease and with thrombin. Several of the most promising compounds are approved drugs.

Except of these two very crucial topics a big diversity of different enzyme inhibitors are presented in this issue. 

Thus, there are reports on inhibitors against parasites such as infections caused by *Fasciola* species, which are widely distributed in cattle and sheep causing significant economic losses, and are emerging as human zoonosis with increasing reports of human cases, especially in children in endemic areas. Triclabendazole, is the only drug of choice for humans treatment that is effective against both the mature and juvenile forms of the parasite [8].A series of twenty-eight quinoxaline 1,4-di-*N*-oxide derivatives we selected from the in-house library aiming to study their ability to inhibit vital cathepsin L enzymes from *Fasciola hepatica.* Most of the compounds are synthetic with only a few examples of natural compounds, namely echinomycin and triostin-A. All these compounds are known as antitubercular, antimalarial, antileishmania, and antichagas agents, among other neglected diseases [9].

In silico screening of a library of natural compounds aiming to search for novel inhibitors of parasitic nematode thymidylate synthase (TS) [10] was also reported. TS is a target of antitumor, antiviral, antifungal, and antiprotozoan chemotherapy. The model of nematode TS three-dimensional (3D) structure and compounds capable to bind/inhibit enzyme were chosen and tested as inhibitors of TS, agents toxic to a nematode parasite model (*C. elegans*) grown in vitro, inhibitors of normal human cell growth and antitumor agent, that affect tumor cell growth. Alvaxanthone was found to be strong TS inhibitor as the anthelminthic drug demonstrating also antiproliferative activity in tumor cells.

Presently, no investigational drug is studied without some contribution of in silico methods.

Dmitriev et al. [11] presented the important role of predictions and accurate assessment of clinically relevant drug-drug interaction (DDIs) for novel drug candidates of drug research and development. It was highlighted that the DDIs should be determined in the early stage of the drug discovery process, according to the requirements of the US Food and Drug Administration (FDA). The danger of strategies usually used by companies was stressed since the developed new chemical entity (NCE) may finally be unacceptable due to the DDIs found in experiments.

One example of the involvement of computational methods in drug design is various virtual screening approaches to identify C1s focused libraries that lack the amidine/guanidine functions and then evaluation by in vitro biological assays. The generation of the pharmacophor model permitted identifying two novel chemotypes with sub-molecular activities. Among 89 compounds tested 20 were identified as hit compounds with the highest activity to be 12 nM. Screening for Factor Xa in term of selectivity measurements, six compounds showed very good selectivity [12].

Another example is the virtual screening of more than 1.7 million of commercially available compounds, which was performed based on the molecular docking results, predicted physico-chemical and ADMET properties and molecular dynamics simulations in order to search for an anticancer drug. As a target Tankyrase enzymes (TNKS) were chosen [13].

The known target for anticancer is also human Protein Kinase (CK2) inhibitors some of which are currently in clinical trials. The natural compound bikaverin, by virtual screening of the ZINC database, was found to be a CK2 inhibitor with an IC50 value of 1.24 μM, fitting well in the ATP binding site of the enzyme [14]. 

Importance was given to the development of an inhibitor of *Mycobacterium tuberculosis* TMPK (*Mtb*TMPK) which is still belong to the ten causes of death worldwide, killing 1.5 million lives in 2018, thereby preceding AIDS. Thus, a series of 1-(1-arylethylpiperidin-4-yl)thymine analogs as *Mycobacterium tuberculosis* TMPK (MtbTMPK) inhibitors based on a 1-(1-(3-phenoxyphenethyl)­piperidin-4-yl)pyrimidine-2,4(1*H*,3*H*)-dione were synthesized and evaluated for their capacity to inhibit MtbTMPK catalytic activity and the growth of a virulent *M. tuberculosis* strain (H37Rv) by Van Calenbergh [15].

Attention was paid to identify novel scaffolds with antifungal activity against *Aspergillus fumigatus,* one of the most ubiquitous fungal pathogen, targeting the fungal cytochrome P450 dependent lanosterol 14-α-demethylase (CYP51A) enzyme. By the combination of *in silico* techniques and in vitro assays, a ligand-based pharmacophore model was created which served as a 3D search query to screen the ZINC chemical database. Molecular docking and molecular dynamic simulations were used to improve the reliability and accuracy of virtual screening. As a result, all tested compounds of different scaffolds exhibited antifungal activity [16]. 

Another topic that is highlighted in this issue is Alzheimer's disease. Pisani et al., aiming to find novel multipotent inhibitors of cholinesterases (acetyl- and butyl-, AcheE BChE) as potential anti-Alzheimer molecules, synthesized and investigated a series of 4-aminomethyl-7-benzyloxy-2*H*-chromen-2-ones, making some modifications around this core [17]. These modifications had positive effects in the case of MAO B activity, while failed with ChEs. The screening revealed a five triple–acting compounds with nanomolar and selective MAO B inhibition and IC50 against AChEs at the low micromolar level. In order to elucidate the possible mechanism of action enzymatic analysis toward AChE as well as docking simulations on the target enzymes were performed.

Among enzyme inhibitors that are discussed in this issue are also inhibitors of coagulation factors Xa and XIa, carbonic anhydrise (CA), K2 and lipoxygenaze (LOX). Thus a series of pyrrolo [3,2,1-ij]quinolin-2(1*H*)-one was synthesized, studied their structure-activity relationships and evaluated their inhibitory activity as inhibitors of coagulation factors Xa and XIa. Four derivatives were able to inhibit both factors Xa and XIa with IC50 of 3.68 μM and 2 μM for the best two Xa and XIa inhibitors respectively [18].

Coumarin/dihydrocoumarin derivatives were evaluated as carbonic anhydrize inhibitors against the cytosolic human isoforms hCA I and II and the transmembrane hCA IX and hCA XII. Two compounds exhibited potent inhibitory activity against hCA IX, showing better or equal potency that acetazolamide used as a reference drug. For the study of the binding mode of this class coumarins/dihydrocoumarins within the active site of hCA IX and XII computational methods were used, validating hCA IX and XII as anti-tumor targets [19].

Novel derivatives of some non-steroidal anti-inflammatory drugs, as well as of the antioxidants α-lipoic acid, trolox and (E)-3-(3,5-di-tert-butyl-4-hydroxyphenyl)acrylic acid with lorazepam were synthesized and evaluated for their anti-inflammatory, LOX and hypolipidemic activity. 

Finally, there are two reviews in this special issue. One dedicated to JAK inhibitors. This review of Alcaro et al. [20] gives an outline of the last four years’ progress in the development of JAKis, mentioning the role of JAKs as a player in multifactorial diseases, the structure of Janus kinases, synthetic strategies for the development of Ruxolitinib, an approved drug against myelofibrosis as well as of Tofacitinib, a drug against rheumatoid arthritis, psoriasis, arthritis and ulcerative colitis. The advantages of enhancing JAKis through the structure-based drug design strategies highlighting the reduction of time and cost by using computational methods were reported.

The second review deals with sodium-glucose co-transporter 2 (SGLT2) inhibitors as a class of anti-hyperglycemic drugs approved for the treatment of type 2 diabetes. In this review, there is a detailed analysis of the SGLT2 inhibitor mechanisms on sodium handling, associated electrolyte abnormalities, evidence of SGLT2i action on sodium transporters, their off-target effects and their potential role on kidney protection as well as their influence on electrolytes and mineral homeostasis [21].
